# Syrup of Dover’s Powder

**Published:** 1879-08

**Authors:** 


### SYRUP OF DOVER'S POWDER.

The officinal preparation of Dover's Powder has been changed into a liquid and a more palatable form. The syrup contains an equivalent of five grains of Dover's Powder to each teaspoonful, and it is found by many of our physicians equally as reliable and efficacious. In the use of an anodyne and expectorant in cough syrups, and as a diaphoretic in febrile disorders, this new form is found to be very convenient for administration and satisfactory in its effects.

---

The following article will be recognized as a very apt and pointed burlesque upon a subject which has more than once deserved ridicule. All medical men, not themselves mechanical geniuses, must have been impressed with the absurdity of the pretensions of many so-called inventions and improvements. The majority are useless; the minority rarely involving a new mechanical principle or a re-adaptation of an old one; the change trivial and unimportant, being nothing more than would occur to the average practitioner in any moment of need. The really valuable inventions are best honored by observing and emphasizing this distinction.

